# CD4^+^ T cell activation promotes the differential release of distinct populations of nanosized vesicles

**DOI:** 10.3402/jev.v1i0.18364

**Published:** 2012-04-16

**Authors:** Els J. van der Vlist, Ger J.A. Arkesteijn, Chris H.A. van de Lest, Willem Stoorvogel, Esther N.M. Nolte-'t Hoen, Marca H.M. Wauben

**Affiliations:** 1 Department of Biochemistry & Cell Biology, Faculty of Veterinary Medicine, Utrecht University, Utrecht,, The Netherlands; 2 Department of Infectious Diseases and Immunology, Faculty of Veterinary Medicine, Utrecht University, Utrecht, The Netherlands

**Keywords:** nanosized vesicles, exosomes, microvesicles, microparticles, extracellular vesicles, flow cytometry, NTA, T cells, immune regulation

## Abstract

Many cell types release nanosized vesicles derived from endosomal compartments (exosomes) or the plasma membrane. Vesicles actively released by CD4^+^ T cells have immune-modulatory characteristics. Using our recently developed high-resolution flow cytometry-based method for the analysis of individual nanosized vesicles, we here investigated how T cell receptor (TCR)-triggering and co-stimulatory signals influence the quantity and characteristics of nanosized vesicles released by CD4^+^ T cells. We found that the number of released nanosized vesicles within the buoyant density range characteristic for exosomes (1.10–1.19 g/ml) was increased by TCR-triggering and that additional co-stimulatory signals had a potentiating effect on vesicle release. However, the increase in the number of released vesicles varied substantially between density fractions within the 1.10–1.19 g/ml range and was highest for the vesicle populations in 1.14 and 1.17 g/ml fractions. Heterogeneity was also observed within the individual density fractions. Based on lipid bilayer fluorescent labelling intensity and light scattering, 3 distinct vesicle subpopulations were identified. One vesicle subpopulation increased significantly more upon T cell activation than the other subpopulations, and this was dependent on high levels of co-stimulation. These data show that T cells release a heterogeneous population of nanosized vesicles and indicate that T cells differentially regulate the release of distinct vesicle subpopulations depending on their activation status.

Cells can release different types of vesicles that are either derived from multivesicular bodies (referred to as exosomes) or shed directly from their plasma membrane (1, 2). The timely release of tailor-made vesicles by cells and their specific recruitment by target cells has led to the emerging concept that cell-derived vesicles may be important vehicles for intercellular communication (2). CD4^+^ T cells are key-players in the initiation and regulation of adaptive immune responses. Similar to antigen-presenting cells (APC), CD4^+^ T cells can release vesicles (3, 4). After release, T cell-derived vesicles can be targeted to different types of immune cells and modify their function. For example, APO2L and FasL-containing T cell vesicles can induce apoptosis of targeted T cells (5–7). T cell-derived vesicles may also block MHC molecules on dendritic cells (DC) or induce apoptosis of DC (8, 9). Furthermore, we have shown that anergic CD4^+^ T cells, in contrast to their non-anergic counterparts, release vesicles that can endow APCs with immune-suppressive properties (10). Besides downregulation of immune responses, T cell-derived vesicles can also induce immune activation. For example, vesicles secreted by activated T cells can bind to monocytes and induce the production of proinflammatory cytokines such as TNFα and IL-1β (11–13). Furthermore, mast cells can be triggered to degranulate and release IL-8 and oncostatin M upon binding of T cell-derived vesicles (14). Altogether these findings indicate that T cell-derived vesicles are targeted to various types of immune cells and are involved in immune regulation at distinct levels.

The number of released vesicles and their molecular composition (proteins, lipids and RNA) is dynamic and dependent on their subcellular origin and the activation status of the producing cell (2). Hence, the total pool of released vesicles can be heterogeneous (15, 16). Vesicles can be characterized based on size, using differential centrifugation and/or filtration, or on buoyant density, using density gradient ultracentrifugation (17). The nanosized exosome population is assumed to be rather homogeneous and has a reported equilibrium buoyant density in sucrose gradients of 1.10–1.19 g/ml (2, 17). Exclusive molecular markers for exosomes or other types of extracellular vesicles have not yet been defined, which complicates the identification of genuine distinct vesicle populations (18). To analyse different vesicle types or subpopulations within heterogeneous vesicle populations, multiparameter high-throughput analysis of individual vesicles is required. Given that the vast majority of cell-derived vesicles is smaller than 300 nm, high-resolution techniques are needed for their visualisation and characterization (19–22). Recently, we developed a high-resolution flow cytometric method to detect, quantify and characterize individual nanosized vesicles based on fluorescence (16). Using this novel method, we were able to identify and analyse the dynamics of different nanosized DC-derived vesicle subpopulations (16).

To understand the pleiotropic roles of CD4^+^ T cell-derived vesicles, it is of utmost importance to identify the triggers for vesicle release by these T cells and to characterize the released vesicle population(s). Previously, it has been postulated that the number of released CD4^+^ T cell-derived vesicles increases upon T cell receptor (TCR)-triggering (3, 7). This was based on an increase of total protein and increased detection of CD63 in pelleted vesicles using Western blotting (3, 7). However, since the molecular composition of the released vesicle population is dynamic (16), protein assays or Western blot analysis of bulk isolates of vesicles are not reliable for quantitative analysis. We here used our high-resolution flow cytometric method to quantify and characterize the population of nanosized vesicles that is released by CD4^+^ T cells in response to different activation stimuli. The individual vesicle-based analysis will be helpful to unravel the physiological role of these vesicles in communication between T cells and other immune cells.

## Materials and methods

### T cell clones and cell culture

The p53-specific CD4^+^ T cell clone (KO4C1) is generated in a C57BL/6 p53 knockout mouse and recognises the peptide corresponding to amino acids 77–96 of murine p53 (23). As previously described (24), T cells were restimulated with peptide-pulsed irradiated splenocytes for 2–3 days and isolated through centrifugation onto Ficoll-Hypaque after which they were expanded with recombinant human IL-2 (Roche, Almere, The Netherlands). T cells were cultured in IMDM (Gibco, Invitrogen, Bleiswijk, The Netherlands) with 10% exosome-free fetal calf serum (FCS; Sigma-Aldrich, Zwijndrecht, The Netherlands), 100 UI/ml penicillin, 100 µM streptomycin, 2 mM Ultraglutamine and 30 µM βmercapto-ethanol (T cell medium) and maintained at 37°C, 5% CO_2_. To deplete FCS from exosomes and other vesicles, 30% FCS in IMDM was ultracentrifuged for at least 15 hours at 100,000*g* (SW28 rotor). For experiments, 10×10^6^ T cells were cultured in 12.5 ml T cell medium supplemented with IL-2 (5 U/ml) in 10 cm dishes for 20 hours. To activate T cells, dishes were coated overnight with 0.1 or 10 µg/ml anti-CD3 (clone 145.2C11) alone or combined with 0.5 or 5 µg/ml anti-CD28 (clone PV-1) in phosphate-buffered saline (PBS) at 4°C. Antibody-coated dishes were washed 3 times with IMDM, and once with exosome-free T cell medium, before T cells were added to the coated plates. For flow cytometric analysis of cells, 4×10^6^ T cells were cultured in a separate 6-cm dishes (coated with the same antibody concentrations) parallel to the cultures in 10-cm dishes for vesicle isolation. T cells in 6-cm dishes were treated with brefeldin A (10 µg/ml) 2 hours prior to antibody labelling to induce intracellular accumulation of interferon-gamma (IFN-γ) (25). Experiments were approved by the institutional ethical animal committees at Utrecht University (Utrecht, The Netherlands).

### Flow cytometric analysis of cells

After 20 hours of culture, including 2 hours of incubation with brefeldin A, cells were harvested and labelled for CD69 and TCR (Vβ11) for 30 minutes on ice in PBS/1% bovine serum albumin (BSA). IFN-γ labelling was performed for 30 minutes on ice, after fixation and permeabilization. Anti–CD69-PE (H1.2F3), anti-TCR-Vβ11-PE (CTVB11), anti-IFN-γ-APC (XMG1.2) and isotype control antibodies were from eBiosciences (Vienna, Austria). Cells were analysed by flow cytometry using a FACSCalibur and CellQuest (BD Biosciences, San Jose, USA) or FCS Express software (De Novo Software, Los Angeles, USA).

### Vesicle isolation and labelling

Vesicles released by 10×10^6^ T cells during 20 hours of culture were used for high-resolution flow cytometric analysis and nanoparticle tracking analysis (NTA). Released vesicles were isolated from culture supernatants by differential steps of (ultra)centrifugation as described previously (22, 26). In short, culture supernatants were cleared from cells by centrifugation at 200*g* and 500*g*. Both steps were performed twice for 10 minutes. Larger debris was removed from supernatant by centrifugation at 10,000*g* for 30 minutes using a SLA-600TC rotor in a Sorvall RC5Bplus centrifuge. Subsequently, vesicles were pelleted for 65 minutes at 100,000*g* using a SW40 rotor in a Beckman Coulter Optima L-90K ultracentrifuge. All centrifugation steps were performed at 4°C. Vesicle pellets derived from 10 ml culture supernatant were resuspended in 20 µl PBS with 0.2% BSA. For all experiments, a stock solution of 5% BSA was used that had been cleared of aggregates by ultracentrifugation at 100,000*g* for at least 15 hours. Resuspended vesicle pellets were labelled with the fluorescent membrane dye PKH67 (7.5 µM; Sigma Aldrich) according to manufacturer's protocol in a total volume of 200 µl. The staining procedure was stopped after 3 minutes by adding 50 µl FCS that was ultracentrifuged for at least 15 hours at 100,000*g*. Vesicles were then mixed with 1.5 ml 2.5 M sucrose, overlaid with a linear sucrose gradient (1.9 M–0.4 M sucrose in PBS) and floated into the gradient by centrifugation using a SW40 rotor in a Beckman Coulter Optima L-90K ultracentrifuge for 16 hours at 192,000*g*. After ultracentrifugation, fractions of 1 ml were collected from the bottom via a capillary pipette connected to the tubing of a peristaltic pump. The densities of the different fractions were determined by refractometry.

### Flow cytometric analysis of nanosized vesicles

The BD Influx™ flow cytometer optimised for high-resolution flow cytometric analysis of individual nanosized vesicles (Becton Dickinson, San Jose, USA) was used for the analysis of vesicles in different sucrose density fractions as described previously (16). In short, the system was triggered on fluorescence signals derived from the PKH67-labelled vesicles. PKH67 was excited with a 488-nm laser and the emitted light was captured by a PMT with a 528/38 filter. Thresholding on this fluorescence channel allowed discrimination between noise events and the particles of interest. A fluorescence threshold was set based on 0.22 µm filtered PBS, allowing an event rate of not more than 6 events per second. Light scattering was measured in straight line with the laser excitation beam with a collection angle of 15°–25° (reduced wide-angle FSC). Light scattering detection was performed in log mode. Samples were run at low pressure (5 PSI on the sheath fluid and 4.2 PSI on the sample) using a 140 µm nozzle. Fluorescent 100- and 200-nm polystyrene beads (yellow-green-fluorescent FluoSpheres, Invitrogen) were used to calibrate the fluorescence, reduced wide-angle FSC and SSC settings on the flow cytometer. Sucrose gradient fractions were diluted 20 times in PBS and vortexed before measurement. Samples were measured at event rates lower than 10,000 events per second. The 3 bottom fractions of the gradient were left out to avoid possible interference by unbound staining reagent in these fractions with the detection of labelled vesicles. Time-based quantitative measurements were performed as described before (16). In short, after 30 seconds of equilibration time, data were acquired for 30 seconds. The sample line was extensively washed in between different samples. Data were acquired using Spigot software version 6.1 (Becton Dickinson). Acquired data were analysed using FCS Express software (De Novo Software).

### Nanoparticle tracking analysis (NTA)

For size determination of the vesicles based on Brownian motion, the NanoSight LM10SH (NanoSight, Amesbury, United Kingdom), equipped with a 532-nm laser was used. The sucrose density fractions were diluted at least 100 times in PBS and vortexed before application into the sample chamber. Samples were applied with sterile syringes until the solution reached the tip of the nozzle. Samples were measured for 90 seconds at 20°C with manual shutter and gain adjustments, which were kept constant for all measurements. The vesicle movement was captured and analysed using the NTA software version 2.2 (NanoSight). Each particle was identified and its Brownian movement tracked and measured frame-to-frame. Based on the particle movement velocity, the particle size was calculated using the Stokes–Einstein equation. Postacquisition settings were optimised and kept constant between all measurements. For each sample, a batch of at least 5 individual movies was captured. For analysis, the tracks (individually traced vesicles) of all movies within 1 batch were used. The number of tracks in each batch analysis was at least 497.

### Statistics

Results are expressed as mean±standard deviation. Significance of the fold increase in number of vesicles in pooled density fractions or per density fraction (as compared to the number of vesicles in the non-activated condition, which was set to 1) was tested with a 1-sample T-test with Bonferonni correction. Differences between multiple groups were compared using the ANOVA, in which the experimental batch was introduced as a random factor, post-hoc comparisons were performed using Tukey's post-hoc test. When a significant interaction was observed between 2 factors (as was the case when the 2 density fractions with respect to the distribution of the 3 subpopulations were compared), both factors were analysed separately. For comparison of the reduced wide-angle FSC geometric means, the paired student's T-test was applied. In all cases a 2-sided p-value <0.05 was considered statistically significant. Asterisks indicate p-values: p < 0.05 (*), p ≤ 0.01 (**) or p ≤ 0.001 (***).

## Results and discussion

In order to quantify and characterize the population of nanosized vesicles released by CD4^+^ T cells after different activation stimuli, T cells were activated by TCR-triggering via plate-bound anti-CD3 in the absence or presence of co-stimulation via plate-bound anti-CD28. With this approach, interactions between T cells and APC were mimicked and T cell-derived vesicles could be analysed in the absence of APC-derived vesicles.

**Fig. 1:**
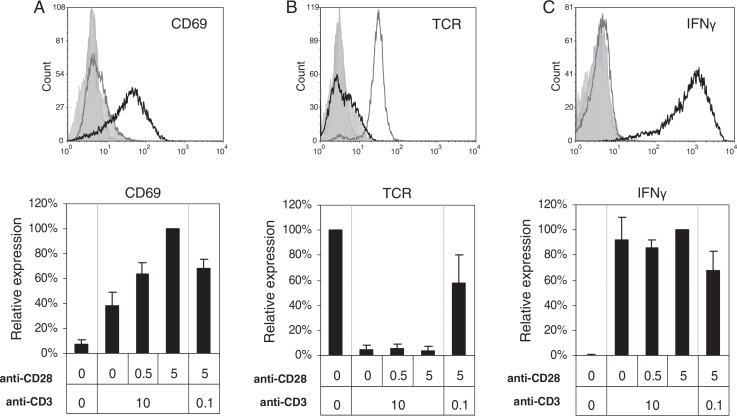
Analysis of T cell activation after different levels of TCR- and co-stimulation triggering. CD4^+^ T cells (KO4C1) were activated by TCR-triggering (0.1 or 10 µg/ml plate-bound anti-CD3), with or without additional co-stimulation (0.5 or 5 µg/ml plate-bound anti-CD28) for 20 hours and compared to non-activated T cells. Cells were analysed for CD69 upregulation (A), TCR downregulation (B) and IFN-gamma production (C) by flow cytometry. Histograms (top panels) show the expression of indicated markers on non-activated T cells (grey line) or on T cells activated with anti-CD3 (10 µg/ml) and anti-CD28 (5 µg/ml) (black line) or isotype control stainings (filled histograms). The bar graphs (bottom panels) show the geometric means expressed as percentages of maximal expression of the indicated markers (set to 100%) of at least 3 independent experiments.

Activation of KO4C1 T cells was confirmed by the expression of the early activation marker CD69, which was highest on T cells activated by high concentrations of both anti-CD3 and anti-CD28 (Fig. 1A). T cells fully downregulated TCR and produced IFN-γ in response to strong TCR-triggering independently of additional co-stimulation (Fig. 1B and 1C). Weaker TCR-triggering in the presence of strong co-stimulation, however, resulted in partial TCR downregulation and lower IFN-γ production (Fig. 1B and 1C).

**Fig. 2:**
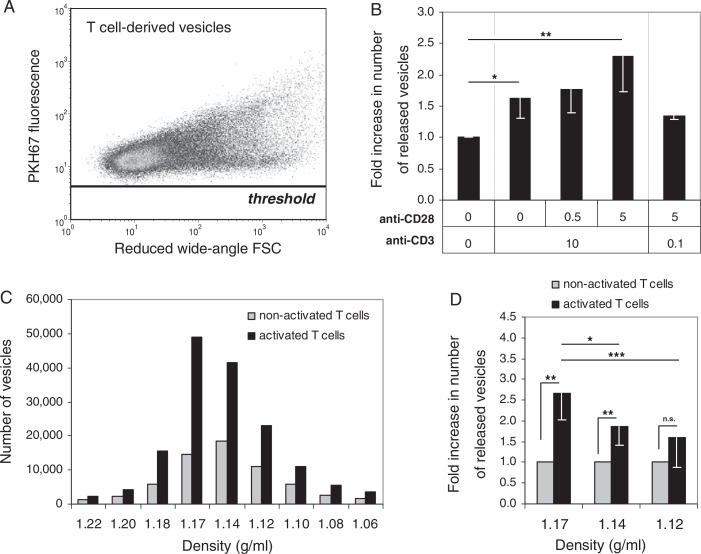
Quantitative analysis of nanosized vesicles released by T cells. KO4C1 T cells were activated as described in Fig. 1. Vesicles released by T cells were isolated from culture supernatants, labelled with the fluorescent membrane dye PKH67 and floated to equilibrium density into a sucrose gradient. The vesicles in the collected sucrose gradient fractions were analysed by fluorescence-based high-resolution flow cytometry. (A) Dot plot of reduced wide-angle FSC versus PKH67 fluorescence representing fluorescent vesicles derived from non-activated T cells (from pooled fractions with densities of 1.12–1.17 g/ml). (B) Fluorescently labelled vesicles from differently activated T cells were quantified using our time-based quantification method. Indicated are the average and standard deviation of the number of vesicles released of at least 3 independent experiments (from fractions with densities of 1.10–1.19 g/ml). The number of vesicles derived from non-activated T cells was set to 1. (C) Fluorescently labelled vesicles from non-activated or activated (10 µg/ml anti-CD3 + 5 µg/ml anti-CD28) T cells were quantified using our time-based quantification method. Indicated is the number of events measured in 30 seconds in the indicated sucrose gradient density fractions. One representative out of 6 experiments is shown (D) Fold increase in the number of released vesicles upon activation (10 µg/ml anti-CD3 + 5 µg/ml anti-CD28) of T cells per density fraction. Indicated are the averages and standard deviations of 9 independent experiments. The number of vesicles released by non-activated T cells was set to 1 for each density fraction. Asterisks denote significant differences (* p<0.05, ** p≤0.01, *** p≤0.001).

To accurately assess the number of nanosized vesicles released by these differentially activated T cells, we used our recently developed fluorescence-based high-resolution flow cytometric method (16). T cell-derived vesicles were collected from cell culture supernatants by differential (ultra)centrifugation, labelled with the fluorescent membrane dye PKH67 and then floated into a sucrose gradient. Nanosized vesicles within the buoyant density range characteristic for exosomes (1.10–1.19 g/ml) were collected and analysed. A threshold was set on fluorescence to distinguish vesicles from noise signals (16). T cell-derived vesicles could be detected well above this threshold (Fig. 2A). As shown in Fig. 2B, KO4C1 T cells that were activated through TCR-triggering released 1.6-fold (±0.3, p = 0.04) more vesicles in comparison to non-activated T cells. These data confirm previous indications based on bulk analyses that the number of nanosized vesicles released by CD4^+^ T cells increases upon TCR-triggering (3, 7). Importantly, we observed that co-stimulation had a potentiating effect on the release of T cell-derived vesicles. Simultaneous strong triggering of TCR and CD28 resulted in a 2.3-fold (±0.6, p = 0.01) increase, as compared to non-activated T cells, whereas strong co-stimulation in the presence of weak TCR-triggering only slightly increased vesicle release (Fig. 2B). Although TCR downregulation and IFN-γ production were not increased by additional co-stimulation in the KO4C1 T cell clone, the potentiating effect on CD69 upregulation indicated that co-stimulation affected the activation status of the T cells. Concomitantly, we observed that the release of vesicles was further increased by co-stimulation.

Previously, we observed that the total pool of nanosized vesicles released by cells can be heterogeneous (16). We, therefore, performed a more detailed quantitative analysis of vesicles that equilibrated at different densities in a sucrose gradient. We found that the vast majority of nanosized vesicles derived from non-activated T cells and T cells activated with high levels of TCR-triggering and co-stimulation equilibrated at densities of 1.12–1.17 g/ml (Fig. 2C). The highest increase in number of released vesicles observed after T cell activation could be attributed to vesicles with a buoyant density of 1.14–1.17 g/ml (Fig. 2D). Moreover, the 2.7-fold increase of vesicles with a buoyant density of 1.17 g/ml was significantly higher compared to the 1.9-fold increase in vesicles equilibrating at 1.14 g/ml (Fig. 2D). These data indicate that heterogeneity exists within the pool of released T cell vesicles and that the release of different vesicle populations is differentially regulated upon T cell activation.

**Fig. 3:**
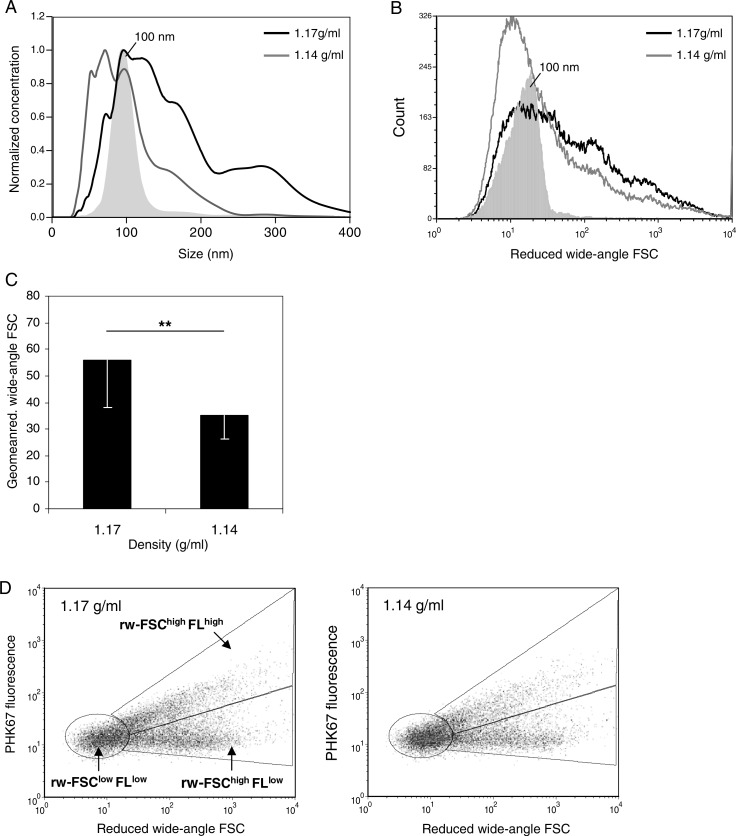
Heterogeneity in T cell-derived vesicle populations with different buoyant densities. Vesicles released by non-activated T cells were isolated and analysed as described in Fig. 2. Histograms indicating size as determined by NTA (A) or reduced wide-angle FSC as determined by high-resolution flow cytometry (B) of 100 nm beads (filled histograms, light grey) and T cell-derived vesicles in fractions with densities of 1.17 g/ml (black line) or 1.14 g/ml (dark grey line). (C) Reduced wide-angle FSC geometric means of vesicles from non-activated T cells with densities of 1.17 or 1.14 g/ml. Indicated are the averages and standard deviation of geometric mean values of 6 independent experiments. Asterisks denote significant differences (**p ≤ 0.01). Histograms in (A) and (B) are from the same experiment, representative of 6 (A) or 5 (B) experiments. (D) Dot plots representing wide-angle FSC and PKH67 fluorescence of vesicles from non-activated T cells floating at 1.17 (left) and 1.14 g/ml (right). Gates were set around vesicles subpopulations with low reduced wide-angle FSC levels and low fluorescence (FSC^low^FL^low^), high(er) reduced wide-angle FSC levels and high(er) fluorescence (FSC^high^FL^high^) or high(er) reduced wide-angle FSC levels and low fluorescence (FSC^high^FL^low^).

To investigate the vesicles equilibrating at 1.14 or 1.17 g/ml in more detail, we first analysed their size by NTA. NTA is a relatively new technique used to determine the size of individually tracked vesicles based on their Brownian motion (27). When compared to other techniques, such as electron microscopy or atomic force microscopy, NTA is currently the most suitable method for absolute sizing of considerable numbers of individual vesicles (20, 28). Based on NTA, we found that the population of vesicles that equilibrated at 1.14 g/ml had an average size of 103±50 nm (Fig. 3A). Compared to a monodisperse 100 nm bead population, these vesicles showed a more heterogeneous size distribution (Fig. 3A). Vesicles present in the higher density fraction (1.17 g/ml) were larger (166±78 nm) and also displayed a broad size distribution (Fig. 3A).

In flow cytometry, forward scattered light (FSC) is often used as a measure for size. However, conventional flow cytometers do not allow detection of particles smaller than 300 nm based on FSC. We recently developed a flow cytometry-based method for analysis of ~100 nm fluorescent particles by optimizing the FSC detection angle and signal-to-noise ratio (16). Although forward scattering of nanosized particles is not only influenced by their size but also by their refractive index, surface roughness, shape and possibly light absorption (29, 30), we previously demonstrated that our reduced wide-angle FSC based analysis allows for approximate and relative sizing of cell-derived nanosized vesicles with comparable composition (16). In contrast to NTA, flow cytometry-based FSC measurements are not hampered by the size heterogeneity of vesicle populations. Moreover, high-resolution flow cytometric analysis allows the simultaneous analysis of multiple parameters on single vesicles, which is needed for the characterization of subpopulations within the total pool of vesicles. By flow cytometric analysis, we found that vesicles equilibrating at 1.17 g/ml displayed higher FSC levels than vesicles equilibrating at 1.14 g/ml (Fig. 3C). The FSC level distributions displayed by the 2 vesicles populations (Fig. 3B) were largely comparable to their size distribution as determined by NTA (Fig. 3A).

The broad size and FSC distribution of vesicles within the 1.14 and 1.17 g/ml density fractions indicated heterogeneity within these vesicle populations. To study this heterogeneity, we analysed fluorescence and reduced wide-angle FSC levels of these vesicles in more detail. Based on their reduced wide-angle FSC (indicated as rw-FSC) and fluorescence signals (FL), we could observe 3 vesicle subpopulations (Fig. 3D). The vesicle subpopulation exhibiting low rw-FSC and low fluorescence (rw-FSC^low^FL^low^) may consist of relatively small vesicles. The population exhibiting higher rw-FSC and fluorescence (rw-FSC^high^FL^high^) may represent vesicles larger in size or aggregates of smaller vesicles. The rw-FSC^high^FL^low^ population may consist of small vesicles (based on the low fluorescence) containing more or other cargo, which induces higher levels of rw-FSC as compared to the rw-FSC^low^FL^low^ vesicle subpopulation. Alternatively, the rw-FSC^high^FL^low^ population may consist of larger vesicles or aggregates that have a distinct lipid bilayer compared to the rw-FSC^high^FL^high^ vesicles and have, therefore, incorporated less fluorescent membrane dye. These data demonstrate that CD4^+^ T cell-derived nanosized vesicle populations with similar buoyant densities are heterogeneous.

**Fig. 4:**
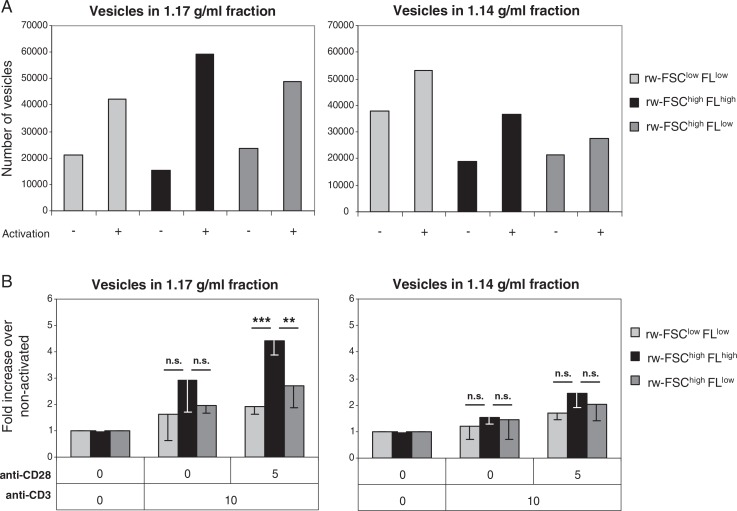
T cells differentially regulate the release of distinct vesicle subpopulations upon different activation signals. Vesicles from activated (10 µg/ml anti-CD3 + 5 µg/ml anti-CD28) and non-activated T cells were isolated and analysed as described in Fig. 2. (A) Time-based quantification of T cell-derived vesicles in different density fractions in the 3 different vesicle subpopulation gates, as described in Fig. 3D. Indicated are the numbers of vesicles per gate measured in 30 seconds. One representative experiment out of 4 is shown. (B) Indicated is the average fold increase ± SD in number of vesicles released by T cells, per FSC-FL gate, activated by TCR-triggering alone or with additional co-stimulation, relative to the number of vesicles from non-activated T cells (set to 1). Averages and standard deviations are displayed for vesicles with a density of 1.17 (left graph) or 1.14 g/ml (right graph) of 4 independent experiments. Asterisks denote significant differences (** p≤0.01, *** p≤0.001).

Next, we investigated whether the 3 subpopulations present within the 1.14 and 1.17 g/ml density fractions (Fig. 3D) similarly changed upon T cell activation. In both density fractions, the number of vesicles in all 3 subpopulations increased upon T cell activation (Fig. 4A). Interestingly, we found a significant difference between the 2 density fractions with respect to the distribution of the 3 subpopulations (p < 0.01). Indeed, the rw-FSC^high^FL^high^ subpopulation present in the 1.17 g/ml fraction consistently increased more upon T cell activation compared to the other subpopulations within this fraction and compared to the subpopulations within the 1.14 g/ml fraction (Fig. 4B). This increase in number of rw-FSC^high^FL^high^ vesicles was dependent on the presence of strong co-stimulatory signals. These data indicate that T cell activation signals can significantly alter the composition of the total pool of released vesicles.

Conclusively, we here show that CD4^+^ T cells release more vesicles upon T cell activation. This increase in vesicle release was most prominent in the vesicle population equilibrating at 1.17 g/ml and was significantly higher compared to other density fractions. Within this density fraction, 1 vesicle subpopulation (FSC^high^FL^high^) increased significantly more as compared to the other subpopulations. This higher increase was significant upon T cell activation with TCR-triggering and additional co-stimulation, indicating that activation signals contribute to the regulated release of distinct vesicle subpopulations. Based on our findings, we hypothesize that APC displaying specific MHC-peptide complexes to T cells may be involved in the fine-tuning of T cell vesicle-mediated intercellular communication by regulating the release of vesicles via co-stimulatory signals. Furthermore, the molecular composition of T cell-derived vesicles might vary depending on the type and level of co-stimulation. It is, therefore, important to study the protein, lipid and RNA content of T cell-derived vesicle populations released after different co-stimulatory triggers. Currently, it is not feasible to isolate vesicle subpopulations by flow cytometric sorting. Alternatively immunoaffinity bead capture of vesicles can be used for selective enrichment or depletion of vesicle subpopulations based on specific protein markers. Furthermore, we recently demonstrated that different vesicle subpopulations with the same buoyant density can be separated by limiting the floatation time into a sucrose gradient (15). The further analysis of the molecular composition of vesicle subpopulations released after different immunological cues will be helpful to determine the physiological role of CD4^+^ T cell-derived vesicles in immune regulation.
